# Assessing the disease burden of non-alcoholic fatty liver disease in the real world – big data and big numbers

**DOI:** 10.1186/s12916-019-1357-y

**Published:** 2019-07-04

**Authors:** Jörn M. Schattenberg, Mattias Ekstedt

**Affiliations:** 1 Department of Medicine, University Medical Center of the Johannes Gutenberg University, Mainz, Germany; 2 Division of Gastroenterology and Hepatology, Department of Medicine and Health Sciences, Linköping University, Linkoping, Sweden

**Keywords:** NAFLD, NASH, Epidemiology, Real-world data, Advanced fibrosis, Fib-4, Awareness, Hepatocellular carcinoma, Screening

## Background

Non-alcoholic fatty liver disease (NAFLD) is a growing burden on European healthcare [1], with estimated annual healthcare costs of €19 billion EUR within the EU-5 region (France, Germany, Italy, Spain and the United Kingdom) [2]. In studies with long-term follow-up, excess mortality in people with NAFLD was shown to be dependent on the degree of underlying hepatic fibrosis [3]. Importantly, NAFLD also significantly impairs patients’ quality of life [4]. The histological definition of both the presence of the inflammatory subtype of the disease, which is commonly called non-alcoholic steatohepatitis (NASH), and hepatic fibrosis, causes challenges for all epidemiological studies on the prevalence of the disease in Europe.

### Evidence from a real-world study

In a recent article published in *BMC Medicine*, Alexander et al. [5] undertook an enormous effort to extract data from the European Medical Information Framework in the UK, Netherlands, Italy, and Spain. They analyzed data from the electronic medical records of more than 18 million European patients with codes for NAFLD and (in some countries) for NASH. The control cohort constituted matched cases with up to 100 non-NAFLD patients for each NAFLD/NASH case and took into account GP practice site, age, sex and healthcare utilization habits into account. This represents one of the most comprehensive and largest control populations; for instance, The Health Information Network covers up to 6.2% of the patients cared for in UK-based GP practices.

The authors confirm established risk factors that are relevant for NALFD; namely type 2 diabetes, arterial hypertension and obesity [6]. Importantly, the study underlines the relevance of these risk factors in a non-referral based, non-high risk population and expands available knowledge through this very large real-world dataset. The authors observed a hazard ratio for cirrhosis of 4.73 (95% CI: 2.43–9.19) and 3.51 for hepatocellular carcinoma (95% CI: 1.72–7.16), underlining the relevance of liver-related outcomes in patients with NAFLD. The incidences of these diagnoses were even higher when the surrogate score for the presence of advanced fibrosis (in this case Fibrosis-4 score [Fib-4]) was ranked as high-risk. While it is likely that a number of NAFLD/NASH cases were not coded in the available healthcare records, this large real-world study cohort highlights the contribution of diabetes as a strong and independent predictor of advanced liver disease and hepatocellular carcinoma, which is in line with previous data and recent data from the USA [7]. Interestingly, the median time between coding liver disease and cirrhosis was, in general, short, ranging between 0.5 and 2.9 years. This highlights shortcomings in the current practice aiming to identify patients with developing liver disease at an early stage.

### Challenges and implications for clinical practice in NAFLD

Considering all available data, including the confirmation arising from this study, the reality of liver disease in metabolically diseased patients is highlighted: diagnosis is made at a late stage. It can take a long time – even several years – to develop advanced liver disease, during which time preventative or even therapeutic approaches could be implemented. However, the fact that many patients are diagnosed at an advanced stage, means that they are prevented from modifying the risk factors responsible for driving the progression of liver disease. This must be highlighted, particularly to primary and secondary care providers. To achieve this, the scientific field urgently needs real-world data from unselected clinical cohorts that represent the disease spectrum of the general population. In the current study, 0.7% of included patients had a diagnosis of NAFLD/NASH; this should be interpreted in light of the 24% estimated global prevalence of NAFLD [8]. Therefore, this study evaluates not the tip of the iceberg, but the tip of the tip of the iceberg.

Although this cohort probably represents a selected subtype of NAFLD/NASH, this study gives us a real world insight into the low awareness of NAFLD/NASH in primary care. We also learn that having a code for NAFLD/NASH does not, in itself, significantly raise awareness of this disease. With the advent of medical therapies for the treatment of NAFLD [9], the minimalistic approach to this patient population will be likely to change. It should also be highlighted that, even in the absence of a liver-directed, specific therapy, NAFLD is an ‘indicator disease’, which identifies patients at risk of developing diabetes [10] or cardiovascular disease and complications [8].

## Conclusions

In summary, this study, together with many others, has clearly established NAFLD/NASH – with its metabolic comorbidities – as a public health challenge. Now it is time to support patients, advocacy groups and specialists to raise awareness about this liver disease.

## Data Availability

Not applicable.

## Abbreviations

NAFLD: Non-alcoholic fatty liver disease
NASH: Non-alcoholic steatohepatitis
